# Supplementary material for: Predicting the influence of extreme temperatures on grain production in the Middle-Lower Yangtze Plains using a spatially-aware deep learning model
Source: PeerJ. 2024 Oct 18;12:e18234. doi: 10.7717/peerj.18234 (PMC11493067; doi:10.7717/peerj.18234)
Supplement: Supplemental Information 1 [file peerj-12-18234-s001.pdf]

Supplementary Articles, Tables and Figures to Predicting the Influence of Extreme Temperatures on Grain Production in the Middle-Lower Yangtze Plains Using a Spatially-Aware Deep Learning Model

Zijun Mu[1] and Junfei Xia[2,3]

[1]Nanjing Smardaten Technologies Co., Ltd, Nanjing, Jiangsu 210000, China
[2]Georgia Institute of Technology, Atlanta, Georgia 30332, United States
[3]Rosenstiel School of Marine, Atmospheric and Earth Science, University of Miami, Miami, Florida 33149, United States

Corresponding author:
Junfei Xia[2,3]

Email address: jxia83@gatech.edu

S1 MODEL TRAINING AND HYPERPARAMETER TUNING

Before training, hwave and cwave data of each case are downscaled to a 50*50 spatial grid with an annual temporal resolution. tas and pr data are downscaled to a 52*52 spatial grid on a monthly temporal resolution. Results of 1-to-1 Pearson Correlation performed for each pair of two variables in the input set are collected in Fig. S1.

In this study, grid search is used to optimize hyperparameters of the sAE, the stAE, and the RF regression. It is implemented by designating a set of candidate values $\mathbf{S}_j$, listed in Table S1, for the j-th hyperparameter. With these sets, the search grid $\mathbf{G_M}$ for model $\mathbf{M}$ is defined as the Cartesian product of the respective sets of all n parameters tuned, i.e.,

$$\mathbf{G_M} = \mathbf{S}_{1,\mathbf{M}} \times \mathbf{S}_{2,\mathbf{M}} \times \cdots \times \mathbf{S}_{n-1,\mathbf{M}} \times \mathbf{S}_{n,\mathbf{M}}$$

S1.1 Convolutional Autoencoder

The ConvAE training/validation set is divided at a 9:1 ratio, and minimum validation loss is computed at each $\mathbf{G}_{\text{sAE}}$ and $\mathbf{G}_{\text{stAE}}$, for the respective climate variable. The hyperparameter combination with which the minimum of validation loss is minimized is selected as the final hyperparameters for stAE:pr, sAE:hwave and sAE:cwave. Due to a different behavior of stAE:tas, the combination at which stAE:tas starts stable and gradually becomes volatile is selected for this model. Training losses over epochs at the optimum learning rate of each ConvAE are plotted on Fig. S3.

sAE and stAE architectures are implemented with PyTorch and trained separately for each meteorological variable of interest. Each ConvAE is trained with 32-sample batches. Various learning rates are experimented with an excessively large number of epochs that guarantees overfitting with the current sample to observe the behavior of the training and validation sets. The optimal result of this experiment is recorded in Fig. S3. Since the resolution of the grid data is not immensely high, 1000 epochs are trained for each ConvAE. For stAE:pr, sAE:hwave and sAE:cwave, the loss is relatively stable across epochs, and the `n_epochs` at which the certain ConvAE's testing Loss is minimized is in the discrete search space $\{x : x \leq 1000, 10|x, x \in \mathbb{N}\}$. As of stAE:tas, whilst the training losses of both the training and testing datasets decline over time, the loss becomes highly volatile after approximately 400 epochs trained. Final training loop hyperparameters used for each ConvAE are listed in Table S2a.

S1.2 Random Forest

For each combination of hyperparameter values in $\mathbf{G}_{\mathrm{RF}}$, model performance is evaluated using k-fold cross-validation. In this study, Mean Absolute Percentage Error (MAPE) will be used as the performance metric. This k-fold cross-validation would seek to minimize mean MAPE of the "folds", i.e., finding a certain $\mathbf{A}^* \in \mathbf{G}_{\mathrm{RF}}$ such that:

$$\mathbf{A}^* = \underset{\mathbf{A}}{\arg\min} \ (\ \mathrm{mean}(\mathrm{MAPE}(E_{n,m})) \ )$$

where $\mathbf{A}^*$ is a combination of hyperparameter values, $E_{n,m}$ the set of predicted values with forest size n at the m-th fold, and MAPE on the set of predicted values $E_{n,m}$, values across the k folds. Therefore, the objective would be to find the value of n that minimizes $\mathrm{mean}(\mathrm{MAPE}(E_{n,m}))$, the mean MAPE of all training-validation set combinations tried in the process.

S2 INFORMATION ON CMIP6 GCM USED

A full list of the 25 GCMs selected and their information is included in Table S5. Taylor (2001) proposed a diagram that statistically summarizes how well geospatial data match each other's patterns in terms of their correlation (such as Pearson correlation) and the ratio of their variances (therefore standard deviations). In this study, to evaluate the performance of downscaled GCMs, standard deviation of each variable and the data's Pearson correlation with the original datasets are plotted onto a polar grid in Fig. S5. Overall, the 25-model ensemble excels at reconstructing historical mean, minimum and maximum temperatures while demonstrating a degree of deviation from the precipitation dataset used, with the ensemble uniformly having a correlation over 0.8 with the observation datasets. However, the models are systemically biased. The standard deviation of monthly mean of historical tas and daily historical tasmax falls uniformly below the observation standard deviation of the respective variable. The daily historical tasmin ensemble has an average (excluding 1 outlier) of approximately 8.6, which is, then, approximately 0.2degC more than the observation standard deviation.

It should be noted that HW frequencies projected in this study show diminishing step-wise increases over time, which can result from underestimation due to the bias of CMIP6 GCMs on climate extremes (Fan et al., 2020). Moreover, Table S6 shows that several commonly used model components are relatively biased within the ensemble. The AOGCM used for ScenarioMIP of models ACCESS-CM2, HadGEM3-GC31-LL, HADGEM3-GC31-MM, KACE-1-0-G, and UKESM1-0-LL are all supported by the Atmos component MetUM-HadGEM3-GA7.1, and all fell in the lower 50% of HW frequency projections. Noteworthily, Among those who utilized MetUM-HadGEM3-GA7.1 as their Atmos component, ACCESS-CM2 (with ACCESS-OM2) and KACE-1-0-G (with MOM4p1) did not use the NEMO Oceanic model, and they represent, in 3 of the four scenarios, the most "extreme" HW frequency projections attained by those within this ensemble. This reaffirms the precise impact of Oceanic components on terrestrial ecosystems and the long-term projected climate. Moreover, UKESM-0-LL, HadGEM3-GC31-LL, and HadGEM3-GC31-MM all used NEMO-HadGEM3-GO6.0 Oceanic components, but HadGEM3-GC31-MM projected significantly more heatwaves than the other two. This reflects the strong impact of the GCM modeling approach on projection results, as illustrated in previous studies (such as Jiang et al. (2020) and Fan et al. (2020)).

REFERENCES

Fan, X., Miao, C., Duan, Q., Shen, C., and Wu, Y. (2020). The performance of cmip6 versus cmip5 in simulating temperature extremes over the global land surface. *Journal of Geophysical Research: Atmospheres*, 125(18).

Jiang, D., Hu, D., Tian, Z., and Lang, X. (2020). Differences between cmip6 and cmip5 models in simulating climate over china and the east asian monsoon. *Advances in Atmospheric Sciences*, 37(10):1102–1118.

Taylor, K. E. (2001). Summarizing multiple aspects of model performance in a single diagram. *Journal of Geophysical Research: Atmospheres*, 106(D7):7183–7192.

LIST OF TABLES

S1 Hyperparameters tested in grid search 4
S2 Final Hyperparameters Used 4
S3 Evaluation statistics of the proposed ConvAE-RF model with benchmark 4
S4 Projected MLYP Total Grain Production (t, 4-year mean across models) 4
S5 Selected CMIP6 GCMs in the NEX-GDDP-CMIP6 Ensemble 5
S6 2-way split of sorted GCMs based on projected HW frequency. 5

Table S1. Hyperparameters tested in grid search

sAE	n_epochs<br>$\{x : x \leq 1000, 10 \vert x, x \in \mathbb{N}\}$	Learning Rate<br>{1e-5, 5e-5, 1e-4, 1.5e-4, 2e-4, 5e-4}	
stAE	n_epochs<br>$\{x : x \leq 1000, 10 \vert x, x \in \mathbb{N}\}$	Learning Rate<br>{1e-5, 2e-5, 4e-5, 8e-5, 1e-4, 1.5e-4, 2e-4}	
RF	n_estimators<br>$\{x : 200 \leq x \leq 750, 25 \vert x, x \in \mathbb{N}\}$	max_depth<br>{None, 10, 20, 30, 40, 50}	
	min_samples_split<br>{2, 4, 6, 8, 10}	min_samples_leaf<br>{1, 2, 4}	ccp_alpha<br>{0.0, 0.001, 0.01, 0.1, 0.2}

Table S2. Final Hyperparameters Used

(a) sAE and stAE

Variable	Batch Size	n_epochs	Learning Rate	Code Size
tas	32	380	8e-5	8
pr	32	390	1e-4	8
hwave	32	210	1.5e-4	8
cwave	32	230	1.5e-4	8

(b) RF Regression

n_estimators	max_depth	min_samples_split	min_samples_leaf	ccp_alpha
500	20	2	1	0.0

Table S3. Evaluation statistics of the proposed ConvAE-RF model with benchmark

Statistic	ConvAE-RF	Benchmark Multilinear FGLS
MAPE	5.984e-02	7.367e-02
EVar	0.9438	0.8721
D	11.77	29.60

Table S4. Projected MLYP Total Grain Production (t, 4-year mean across models)

	2021-2024				2049-2052				2097-2100			
Experiment	Median	Q1	Q3	IQR	Median	Q1	Q3	IQR	Median	Q1	Q3	IQR
SSP126	14589.491	14388.346	14828.931	440.585	14482.063	14332.708	14787.149	454.442	14481.397	14366.355	14723.664	357.309
SSP245	14606.227	14414.940	14835.195	420.255	14540.321	14358.505	14855.511	497.006	14499.167	14277.950	14782.385	504.434
SSP370	14584.645	14390.850	14850.843	459.993	14501.817	14347.967	14786.761	438.794	14489.700	14178.701	14843.262	664.560
SSP585	14641.818	14397.457	14832.825	435.368	14487.352	14312.725	14821.124	508.399	14371.273	14127.815	14539.829	412.015

Table S5. Selected CMIP6 GCMs in the NEX-GDDP-CMIP6 Ensemble

Model	Institution	Atmos Component	Ocean Component
ACCESS-CM2	CSIRO-ARCCSS	MetUM-HadGEM3-GA7.1	ACCESS-OM2
ACCESS-ESM1-5	CSIRO	HadGAM2	MOM4
BCC-CSM2-MR	BCC	BCC-AGCM3-MR	MOM4
CanESM5	CCCma	CanAM5	NEMOv3.4.1
CMCC-CM2-SR5	CMCC	NEMO3.6	NEMOv3.6
CMCC-ESM2	CMCC	NEMO3.6	NEMOv3.6
CNRM-CM6-1	CNRM-CERFACS	Arpege 6.3	NEMOv3.6
CNRM-ESM2-1	CNRM-CERFACS	Arpege 6.3	NEMOv3.6
EC-Earth3	EC-Earth-Consortium	IFS cy36r4	NEMOv3.6
FGOALS-g3	CAS	GAMIL3	LICOM3.0
GFDL-CM4	NOAA-GFDL	GFDL-AM4.0.1	GFDL-OM4p25
GISS-E2-1-G	NASA-GISS	GISS-E2.1	GISS Ocean
HadGEM3-GC31-LL	MOHC	MetUM-HadGEM3-GA7.1	NEMO-HadGEM3-GO6.0
HadGEM3-GC31-MM	MOHC	MetUM-HadGEM3-GA7.1	NEMO-HadGEM3-GO6.0
INM-CM4-8	INM	INM-AM4-8	INM-OM5
INM-CM5-0	INM	INM-AM5-0	INM-OM5
IPSL-CM6A-LR	IPSL	LMDZ	NEMO-OPA
KACE-1-0-G	NIMS-KMA	MetUM-HadGEM3-GA7.1	MOM4p1
KIOST-ESM	KIOST	GFDL-AM2.0	GFDL-MOM5.0
MIROC6	MIROC	CCSR AGCM	COCO4.9
MIROC-ES2L	MIROC	CCSR AGCM	COCO4.9
MRI-ESM2-0	MRI	MRI-AGCM3.5	MRI.COM4.4
NESM3	NUIST	ECHAM v6.3	NEMOv3.4
NorESM2-LM	NCC	CAM4-Oslo	MICOM
UKESM1-0-LL	NIMS-KMA	MetUM-HadGEM3-GA7.1	NEMO-HadGEM3-GO6.0

Table S6. 2-way split of sorted GCMs based on projected HW frequency.

Split	SSP126	SSP245	SSP370	SSP585
lower	INM-CM5-0	INM-CM5-0	INM-CM5-0	NESM3
	INM-CM4-8	INM-CM4-8	INM-CM4-8	INM-CM4-8
	NESM3	NESM3	KACE-1-0-G	INM-CM5-0
	KACE-1-0-G	KACE-1-0-G	UKESM1-0-LL	KACE-1-0-G
	IPSL-CM6A-LR	UKESM1-0-LL	ACCESS-ESM1-5	HadGEM3-GC31-LL
	ACCESS-ESM1-5	ACCESS-ESM1-5	EC-Earth3	ACCESS-ESM1-5
	UKESM1-0-LL	HadGEM3-GC31-LL	MIROC-ES2L	EC-Earth3
	KIOST-ESM	IPSL-CM6A-LR	IPSL-CM6A-LR	ACCESS-CM2
	HadGEM3-GC31-LL	CMCC-CM2-SR5	GISS-E2-1-G	IPSL-CM6A-LR
	EC-Earth3	KIOST-ESM		KIOST-ESM
	MIROC-ES2L	EC-Earth3		
		ACCESS-CM2		
higher	ACCESS-CM2	MIROC-ES2L	ACCESS-CM2	GISS-E2-1-G
	GISS-E2-1-G	GISS-E2-1-G	CNRM-ESM2-1	CanESM5
	CNRM-ESM2-1	NorESM2-LM	CanESM5	GFDL-CM4
	FGOALS-g3	GFDL-CM4	FGOALS-g3	MIROC-ES2L
	CanESM5	CNRM-ESM2-1	NorESM2-LM	NorESM2-LM
	NorESM2-LM	FGOALS-g3	MRI-ESM2-0	CNRM-ESM2-1
	MRI-ESM2-0	CanESM5	BCC-CSM2-MR	FGOALS-g3
	HadGEM3-GC31-MM	CMCC-ESM2	CNRM-CM6-1	BCC-CSM2-MR
	BCC-CSM2-MR	MRI-ESM2-0	MIROC6	CNRM-CM6-1
	CMCC-ESM2	BCC-CSM2-MR	CMCC-ESM2	MIROC6
	CNRM-CM6-1	CNRM-CM6-1		
	MIROC6	MIROC6		

■: Atmos Component: MetUM-HadGEM3-GA7.1.

LIST OF FIGURES

S1 Pearson Correlation between Input Variables in the Dataset Used for Training & Testing of the RF Regressor. Each value in the code of a meteorological variable is listed in a distinct column (order-aware) and applied Pearson correlation accordingly. 7
S2 Two Proposed ConvAE Model Architectures 8
S3 ConvAE Training Loss (Mean Squared Error) at Various Epochs. Each panel of the grid is labeled [architecture]:[varname] @ LR [learning rate], and present training loss (not standardized) on a logarithmic Scale. Selected number of epochs training is indicated by the dotted line. 9
S4 ConvAE-RF Expected and Observed Grain Output for Each MLYP Province, in tons. In the observation scatter, the darker dots are members of the training dataset while the lighter are in the testing set. The line represents the expected grain production predicted by the regression. 9
S5 Taylor Diagram of CMIP6 Historical Model Reconstructions of Meteorological Variables in MLYP through 1980-2014. 10
S6 Extended map of CMIP6 GCM projected ETE frequencies in the MLYP provinces, including current observations, SSP126 temporal range means, SSP245 temporal range means, SSP370 temporal range means, SSP585 temporal range means, and temporal Pearson correlations plotted for each point on the spatial grid. 11

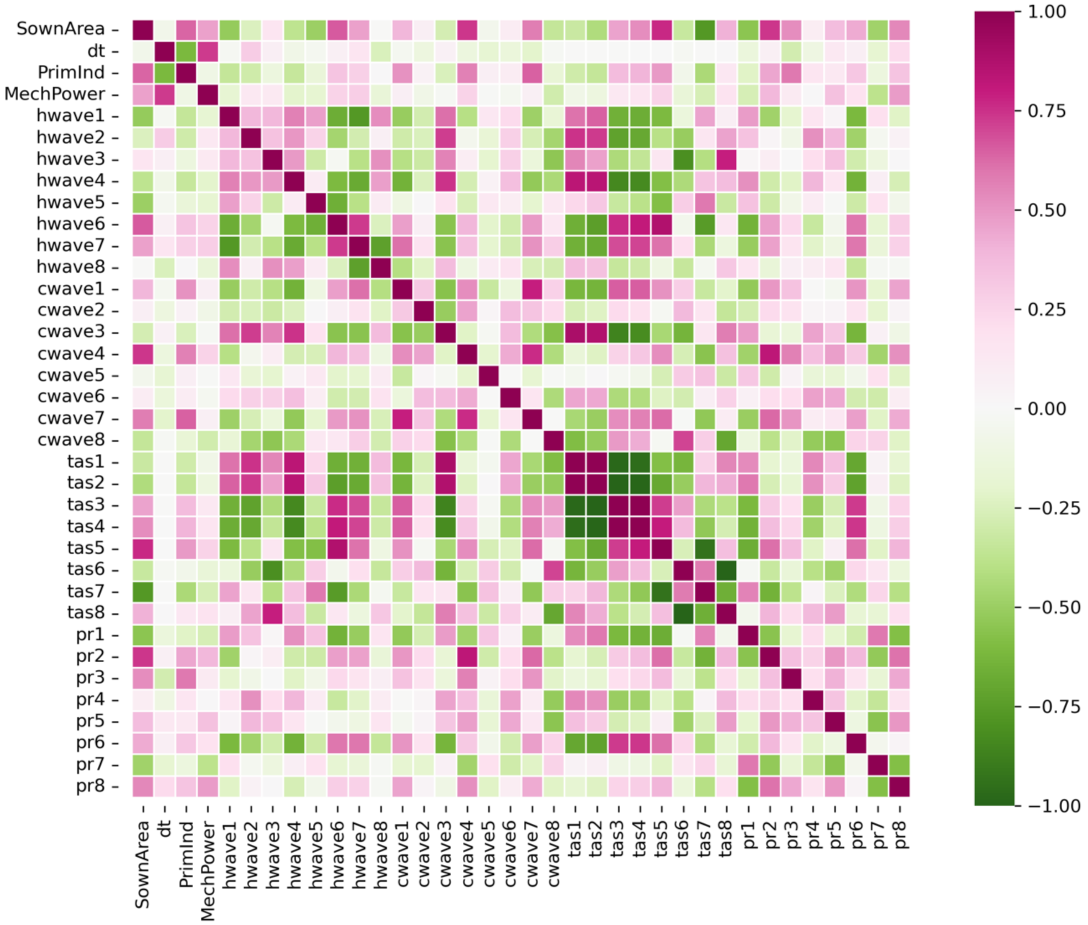

Figure S1. Pearson Correlation between Input Variables in the Dataset Used for Training & Testing of the RF Regressor. Each value in the code of a meteorological variable is listed in a distinct column (order-aware) and applied Pearson correlation accordingly.

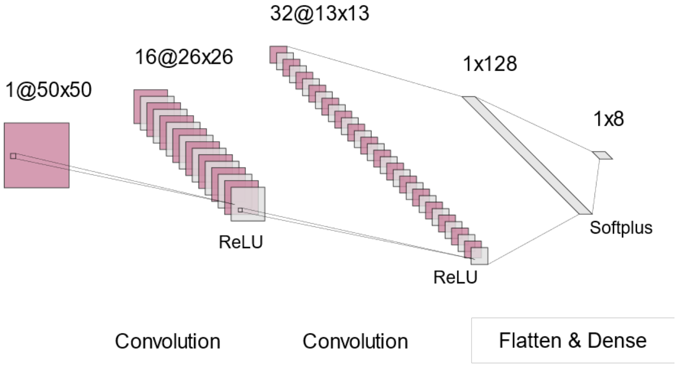

(a) sAE Model Architecture

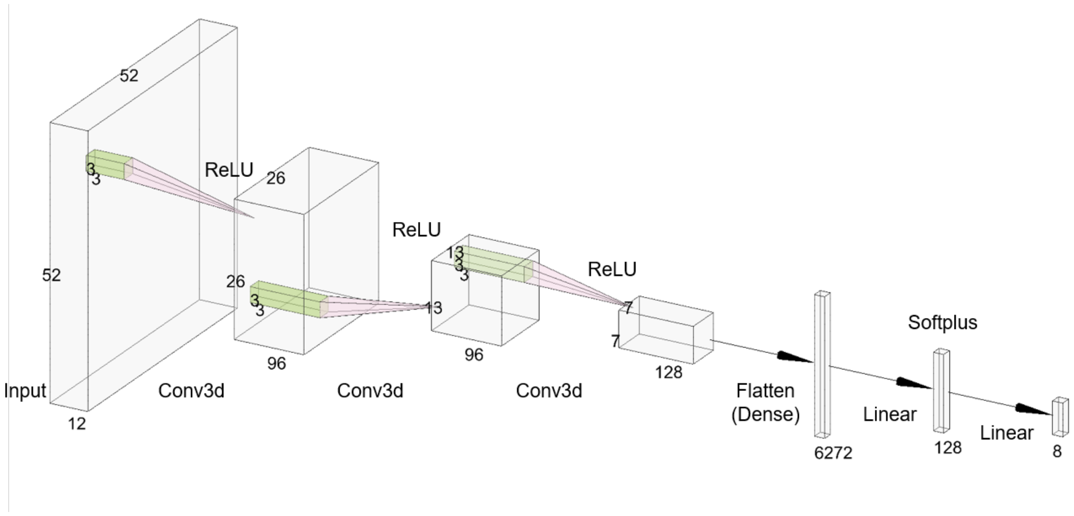

(b) stAE Model Architecture

Figure S2. Two Proposed ConvAE Model Architectures

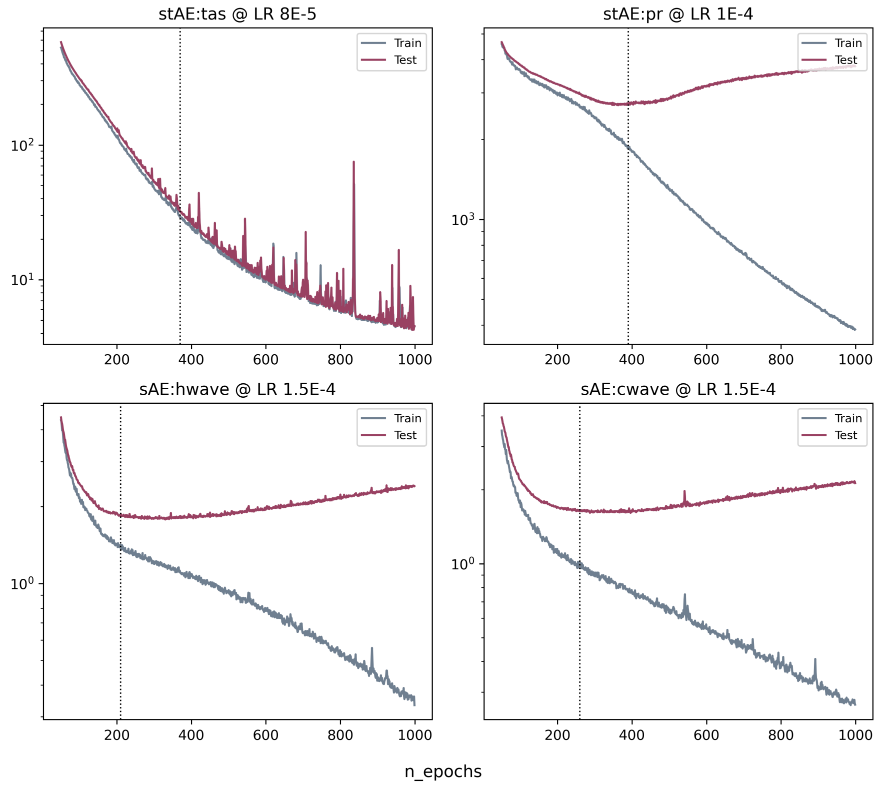

Figure S3. ConvAE Training Loss (Mean Squared Error) at Various Epochs. Each panel of the grid is labeled [architecture]:[varname] @ LR [learning rate], and present training loss (not standardized) on a logarithmic Scale. Selected number of epochs training is indicated by the dotted line.

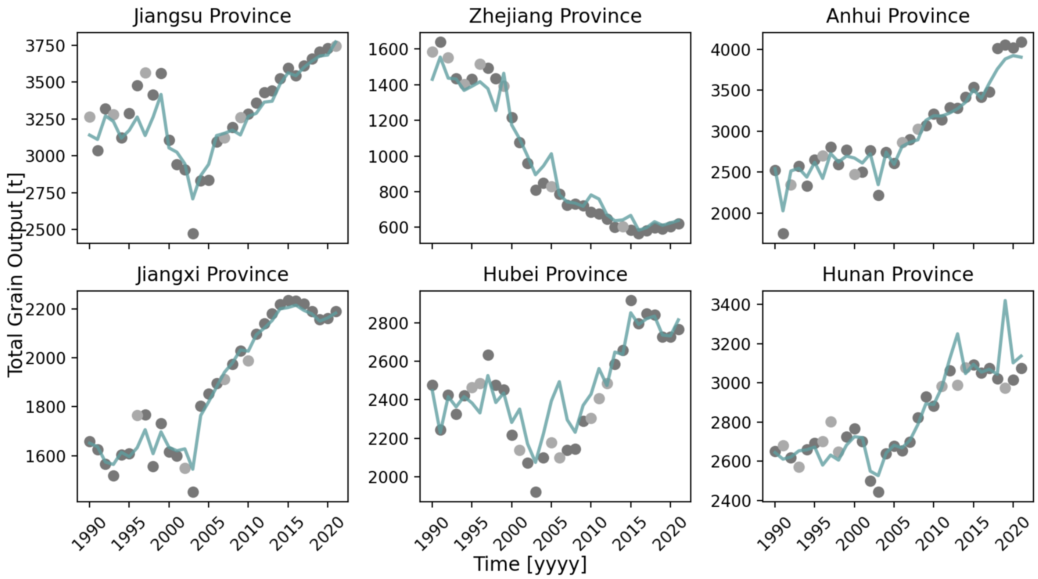

Figure S4. ConvAE-RF Expected and Observed Grain Output for Each MLYP Province, in tons. In the observation scatter, the darker dots are members of the training dataset while the lighter are in the testing set. The line represents the expected grain production predicted by the regression.

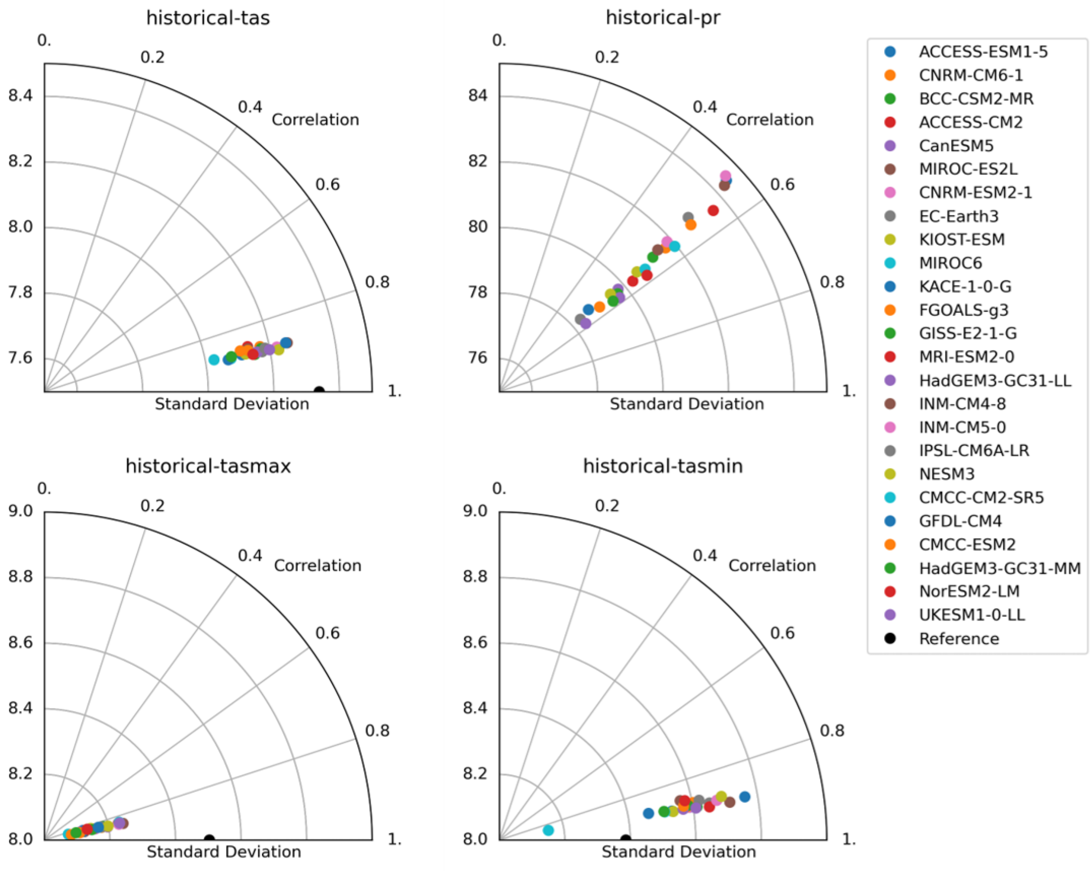

Figure S5. Taylor Diagram of CMIP6 Historical Model Reconstructions of Meteorological Variables in MLYP through 1980-2014.

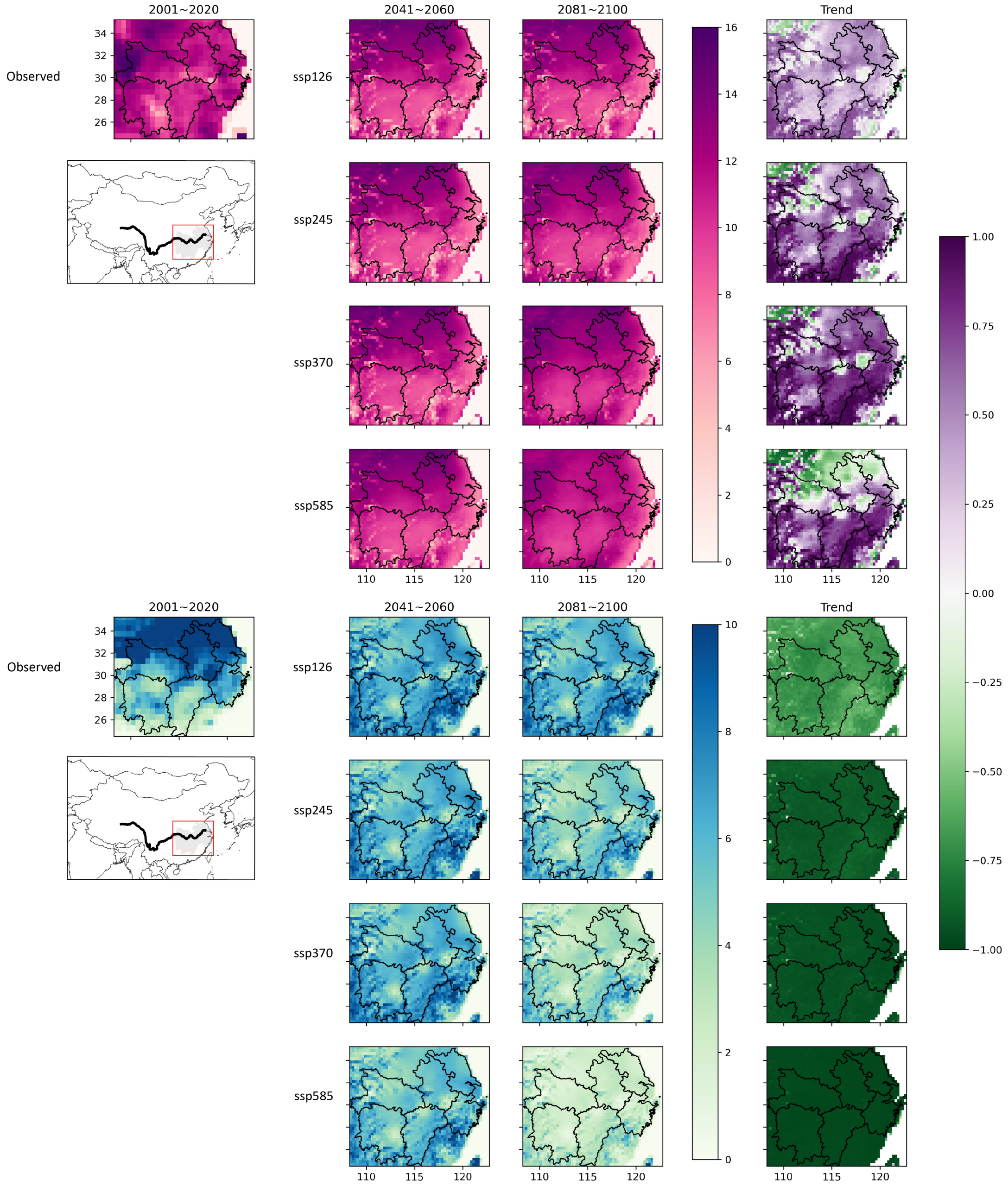

Figure S6. Extended map of CMIP6 GCM projected ETE frequencies in the MLYP provinces, including current observations, SSP126 temporal range means, SSP245 temporal range means, SSP370 temporal range means, SSP585 temporal range means, and temporal Pearson correlations plotted for each point on the spatial grid.
